# Markers of aging: Unsupervised integrated analyses of the human plasma proteome

**DOI:** 10.3389/fragi.2023.1112109

**Published:** 2023-02-22

**Authors:** L. Coenen, B. Lehallier, H. E. de Vries, J. Middeldorp

**Affiliations:** ^1^ Department of Neurobiology and Aging, Biomedical Primate Research Centre, Rijswijk, Netherlands; ^2^ Department of Molecular Cell Biology and Immunology, Amsterdam Neuroscience, Amsterdam UMC location Vrije Universiteit Amsterdam, Amsterdam, Netherlands; ^3^ Alkahest Inc, San Carlos, CA, United States

**Keywords:** aging, proteomics, blood, health, disease, plasma, SomaScan

## Abstract

Aging associates with an increased susceptibility for disease and decreased quality of life. To date, processes underlying aging are still not well understood, leading to limited interventions with unknown mechanisms to promote healthy aging. Previous research suggests that changes in the blood proteome are reflective of age-associated phenotypes such as frailty. Moreover, experimentally induced changes in the blood proteome composition can accelerate or decelerate underlying aging processes. The aim of this study is to identify a set of proteins in the human plasma associated with aging by integration of the data of four independent, large-scaled datasets using the aptamer-based SomaScan platform on the human aging plasma proteome. Using this approach, we identified a set of 273 plasma proteins significantly associated with aging (aging proteins, APs) across these cohorts consisting of healthy individuals and individuals with comorbidities and highlight their biological functions. We validated the age-associated effects in an independent study using a centenarian population, showing highly concordant effects. Our results suggest that APs are more associated to diseases than other plasma proteins. Plasma levels of APs can predict chronological age, and a reduced selection of 15 APs can still predict individuals’ age accurately, highlighting their potential as biomarkers of aging processes. Furthermore, we show that individuals presenting accelerated or decelerated aging based on their plasma proteome, respectively have a more aged or younger systemic environment. These results provide novel insights in the understanding of the aging process and its underlying mechanisms and highlight potential modulators contributing to healthy aging.

## 1 Introduction

The world population has rapidly increased in age from an average lifespan of 45 years in the 1950s to over 70 years nowadays (United Nations, Department of Economic and Social Affairs, Population Division, 2022). During the last two decades the global lifespan increased from 66.8 years in 2000 to 73.4 years in 2019, whereas the healthspan in this period increased at a slower pace from 58.3 to 63.7 years (Organization, 2019). This increasing gap between the life- and healthspan suggest that we live longer, but with a lower quality at the later stages of life. Thus, understanding which processes underly aging may provide valuable insights and possibilities to promote healthy aging, thereby improving quality of life at higher ages.

Aging is associated with changes in the cardiovascular and muscular system (Trombetti et al., 2016; Steenman and Lande, 2017) and with decreased cognitive performance (Bettio et al., 2017). Current evidence also points to an important role of the immune system in aging (Muller et al., 2019). Molecular signatures of such age-associated changes have been found in the blood, the most important transport system connecting the organs in our body. For example, inflammatory components such as cytokines (Milan-Mattos et al., 2019), disease-associated molecules or pathogens are often elevated at later ages (Walker et al., 2022), leading to a chronic state of low-grade inflammation known as ‘inflammaging’. These alterations play a role in multiple age-associated diseases, such as cancer (Huang et al., 2015), cardiovascular diseases (Ferrucci and Fabbri, 2018) and several neurodegenerative diseases (Costantini et al., 2018). Together, these results indicate that changes in blood composition may reflect age-associated changes throughout the body and can provide valuable insights in ongoing biological and disease-related processes.

Several studies showed that defining blood component changes, including altered circulating proteins, provides fundamental insights into numerous diseases (Luczak et al., 2015; Cao et al., 2020; Harbaum et al., 2022) and helps to identify clinical biomarkers and potential therapeutic targets (Hoogeveen et al., 2020; Shi et al., 2021). Using human plasma proteomic data, statistical models termed ‘clocks’ have been developed which accurately predict chronological age (Tanaka et al., 2018; Lehallier et al., 2019). Moreover, plasma proteomic clocks can accurately predict phenotypes such as frailty (Sathyan et al., 2020a; Tanaka et al., 2020) and mortality (Sathyan et al., 2020b), suggesting that the plasma proteome reflects a state of biological functioning. Interestingly, individuals with a lower estimated biological proteomic age compared to their chronological age performed better on several phenotypes such as cognitive and physical tests (Lehallier et al., 2019).

Together, these studies not only promote the idea that changes in the plasma proteome harbor predictive information on aging, but also that modulating it may increase the healthspan. Previous experiments with mice revealed that a shared blood circulation of a young and aged mouse decreased the lifespan of the young mice (Yankova et al., 2022). Moreover, an aged systemic environment was associated with decreased neurogenesis and impaired cognitive performance in young mice (Villeda et al., 2011) and induced a more aged transcriptomic profile across different cell types (Palovics et al., 2022). Conversely, injections of young mouse plasma or plasma from the human umbilical cord rejuvenated several tissues of old mice such as the kidneys, brain and heart and improved their cognitive performance (Loffredo et al., 2013; Katsimpardi et al., 2014; Sinha et al., 2014; Villeda et al., 2014; Baht et al., 2015; Castellano et al., 2017; Huang et al., 2018; Palovics et al., 2022). Recently, a small safety study in elderly people who were injected with human umbilical cord plasma showed that this approach is safe and beneficially altered multiple biomarkers (Clement et al., 2022). These results highlight that factors present in aged blood promote aging and that modulating the blood composition can be a therapeutic option to promote healthy aging in humans.

To date, it remains unknown which proteins contribute to these protective or deteriorating effects during aging. A conserved plasma proteomic aging signature between human and mice has been described (Lehallier et al., 2019), suggesting similar age-associated pathways across species. While most studies highlight a variety of unique potential protein candidates, only few integrated the results across cohorts (Johnson et al., 2020; Lehallier et al., 2020). As many biological and technical factors may influence the plasma proteome, it is important to focus on similar effects across studies to provide stronger evidence for age-associations across proteins in the plasma. Additionally, another limitation is often a relative low number of described proteins due to the lack of overlap between measured proteins across studies resulting of different methods.

To come to a preserved human plasma aging proteome, we here integrated four large-scaled plasma proteome datasets using SOMAscan proteomic assays on independent human cohorts (Sathyan et al., 2020b; Arthur et al., 2021; Ferkingstad et al., 2021; Robbins et al., 2021). These studies each measured ∼5,000 plasma proteins, with a combined age range of 16 to almost 100 years in cohorts which varied in health status from disease free to several comorbidities. Unsupervised integration based on similar aging effects of these datasets resulted in a highly preserved human plasma proteomic aging signature, which is strongly associated to diseases. By comparing proteomic profiles of individuals deviating from their chronological age based on their proteomic plasma profile, we highlight markers of aging and potential modulators which may contribute to a healthier aging process.

## 2 Materials and methods

### 2.1 Identification of aging proteins

Aging proteins were identified by integrating the information of four independent studies using the SOMAscan platform for proteomic measurements (Sathyan et al., 2020b; Arthur et al., 2021; Ferkingstad et al., 2021; Robbins et al., 2021). Two studies stated that they used SomaScan version 4 (Sathyan et al., 2020b; Ferkingstad et al., 2021) and two studies reported a ‘5k assay’ (Arthur et al., 2021; Robbins et al., 2021). As several SOMAmers target similar proteins this provides a resolution on proteoform level, however for readability we refer to ‘proteins’ across this study and an overview of the number of included aptamers across studies is presented in Table 1. Two studies provided raw SOMAscan data of the plasma proteome (Arthur et al., 2021; Robbins et al., 2021), while the other two provided summary statistics of their analyses for all measured plasma proteins (Sathyan et al., 2020b; Ferkingstad et al., 2021). For a full overview of the included studies and demographics, see Table 1. For the studies providing raw data, we performed linear modeling to test for the effect of age on protein expression levels, while correcting for most of the available metadata to correct for possible confounding effects. Proteins were defined as significantly associated to aging at a Benjamini-Hochberg (FDR) adjusted *p*-value (q) below 0.01 (q < .01).

**TABLE 1:** Overview of included study cohorts for identification of APs and validation steps. The cohort name, accessibility to raw data or only summary statistics from their analyses, demographics on age and sex of the cohorts, used coagulant in the studies, source material, health status and number of measured Somamers are provided. Used models or a description of the model is provided which is used to estimate age effects for each protein individually across each study.

	Arthur et al., 2021	Robbins et al., 2021	Sathyan et al., 2020b	Ferkingstad et al., 2021	Sebastiani et al., 2021	Sullivan et al., 2021
Cohort name	ABF300	HERITAGE	LonGenity	deCODE	New England Centenarian Study	COVIDome
Type of Data	Raw data	Raw data	Summary statistics	Summary statistics	Summary statistics	Raw data
Sample material	Plasma	Plasma	Plasma	Plasma	Serum	Plasma
Data usage	Identification of APs	Identification of APs	Identification of APs	Identification of APs	Comparison of age effects	Validation of proteomic clocks
Anticoagulant	Heparin	EDTA	EDTA	EDTA	Not described	EDTA
n (% female)	150 (17%)	745 (55%)	1,025 (56%)	35,559 (57%)	142 (51%)	29 (41%)
mean Age [SD; range]	49 [16.7; 25—80]	34 [13.4; 16–66]	75.8 [6.7; 65-95]	55 [17; range not provided]	Centenarians: 105.7 (SD = 3.6), Controls: 70.6 (SD = 7.8)	45 [16.65; 22–80]
Health Status	Healthy participants, diagnosis established using a screening questionnaire	Healthy, but sedentary over the previous 3 months. Free from cardiometabolic disease	Several comorbid conditions, such as stroke, diabetes and hypertension are present among the cohort	Combined cohort from the Icelandic Cancer Project, enriched for cancer patients, and the deCODE Health Study, which contained several cancer cases and a wide variety of disease phenotypes across the cohort	Centenarians are healthy, controls are not clearly described	Hospitalized, COVID-negative
Model	Protein ∼ Age + Gender + BMI	Protein ∼ Age + Gender + Ethnicity + BMI	Protein ∼ Age + Gender + Cohort	Age effects were estimated using a random-effects model	ANOVA, adjusted by sex and year of sample collection	
# Somamers	5,284	4,977	4,265	5,284	4,785	4,843

### 2.2 Identification of preserved aging protein signature

We calculated FDR adjusted *p*-values (q) for all datasets independently and we overlapped all significant proteins from our four studies based on the provided UniProt identifier. Due to variability in cohorts and the variation in measured proteins across studies, we identified all proteins significantly associated with age in three or more studies showing similar aging effect directions across the studies they were measured in to be our preserved set of Aging Proteins (APs).

### 2.3 Protein-protein interaction networks

We obtained Protein-Protein interaction (PPI) networks using Cytoscape v3.9.1 (Shannon et al., 2003) to mine the String Database (Szklarczyk et al., 2019). Proteins were mapped based on their gene symbol. A high confidence interaction score was used (0.90) for the network creation.

### 2.4 Cluster analysis of the plasma proteome

To cluster the plasma proteome based on similar expression trajectories across aging, we first smoothened the data using a local regression analysis (*loess* function) from the R *stats* package (v4.1.0) with a span of 0.75. For this analysis, we used the proteomic data of Arthur et al. (2021) (Arthur et al., 2021), as this dataset provided the largest age range. The relative fluorescent units (RFU) indicative of protein expression was first log2 transformed, and then z-scores were computed for each protein individually. Next, we applied our LOESS model for each protein separately to reduce noise and variability using the following model: Protein expression ∼ Age *.*

An unsupervised hierarchical clustering analysis was performed using the *hclust* function from the R *stats* package using the ‘complete’ method and a dissimilarity cut-off value of 6.

### 2.5 Enrichment of APs among plasma proteome clusters

To test for enrichments of our APs among our defined clusters, we conducted a hypergeometric test for each cluster individually using the *phyper* function from the *stats* package in R. For each cluster individually, we calculated the probability of obtaining the same number or more of APs for the corresponding cluster size, given the number of Aging Proteins (273 APs) in our full proteomic background dataset (5,284 proteins, as provided by Arthur et al. (2021) (Arthur et al., 2021)). Obtained *p*-values were adjusted using the FDR (Benjamini-Hochberg) method and considered significant at an adjusted *p*-value < .05.

### 2.6 Functional enrichment of APs

To identify the biological relevance of our protein subsets of interest, we mined the KEGG-, GO- and Reactome databases using the R packages *clusterProfiler* (v4.2.2) and *ReactomePA* (v1.38.0). We used Entrez or UniProt identifiers as input and used all 5,284 measured SOMAmers by Arthur et al. (2021) (Arthur et al., 2021) as background set to test for over-representations. SOMAmers mapped to multiple proteins were excluded in these analyses. Using the Benjamini-Hochberg approach, *p*-values were adjusted. Enrichment was defined at a significance level of q < .05. For the GO-analysis, q-values were calculated for each class separately (molecular function, cellular component, and biological process). We modified the parameter setting to the minimum number of genes per category as three.

### 2.7 Associations between APs and phenotypes

To test which phenotypes are enriched in associations with APs, we made use of summary statistics as provided by Ferkingstad et al. (2021) (Ferkingstad et al., 2021). In short, they identified across 373 phenotypes which of their 5,284 plasma proteins were associated to this phenotype after correction for age and sex effects and accounting for multiple testing using the Bonferroni correction. Applying this information of sets of proteins associated to specific phenotypes, we could then infer the number of APs associated to each phenotype. Using a hypergeometric test, we then tested for each phenotype if the number of APs associated to it was greater than expected in the corresponding set size, given the number of APs (273) in the complete proteomic background dataset (5,284 proteins). Nominal *p*-values were corrected using a Bonferroni approach, and associations were considered significant at an adjusted *p* < .01.

### 2.8 Age prediction using the plasma proteome

To determine whether our APs can predict chronological age, we fitted LASSO models (Alpha = 1, minimum lambda value as estimated after 10-fold cross validation) using the R package *glmnet* (v4.1-4) in the available datasets using all 273 APs and sex as input variables. For Arthur et al. (Arthur et al., 2021) we selected 100 individuals to train our model to predict chronological age, which we tested on the remainder of the sample (*n* = 50). For Robbins et al. (Robbins et al., 2021) we selected 500 individuals as training set and tested on the remainder (*n* = 245). Our input variables consisted of the within sample z-scaled log2 transformed APs and gender. To estimate the predictive validity of our models, we correlated for each model the original age with the predicted proteomic age using Spearman’s correlation.

To obtain a reduced model of our APs, we calculated across all models the frequency of each selected input variable. Next, we overlapped the variables used in the majority of the models (selected in > 5,000 models) across both datasets to identify key proteins across datasets. Using Ridge Regression analysis (alpha = 0), we repeated our age prediction as described above using only these 15 proteins and sex as input.

### 2.9 ΔAge estimations

To obtain unbiased estimates and correct for potential under- or overestimates of the predicted proteomic age caused by the fitted proteomic LASSO model, we performed a correction to account for this as described by De Lange & Cole (de Lange and Cole, 2020). In short, after predicting our age using a fitted proteomic model, we fitted a novel linear model to estimate the chronological age (linear model LM=Predicted Age ∼ Chronological Age ) and again predicted the ages of our samples using the following equation:

$$Unbiased\;Age\;Prediction=\left(Predicted\;Age\;–\;Intercept\;of\;LM\right)\;/\;Coefficient\;of\;LM$$

This unbiased estimate of predicted age was then used to subtract from the chronological age to obtain our unbiased ΔAge. Mean average error (MAE) was calculated for each dataset individually as followed:

$$MAE=\Sigma \left(\left|\Delta Age\right|\right)/number\;of\;\Delta Age\;estimates$$

### 2.10 Identification and comparison between accelerated, decelerated and chronological agers

To identify Accelerated Agers (AA), Decelerated Agers (DA), and Chronological Agers (CA) we took the average ΔAge which was calculated across all LASSO models. As random permutations were performed to select 1/3rd of the sample randomly for estimation of the chronological age, participants had ∼3,300 ΔAge estimates, although slight differences in the number of estimates may occur resulting of the random permutations. ΔAge cut-off values were based on the MAE for the models across datasets, and deviating individuals were picked beyond this MAE, whereas non-deviating individuals well within the MAE. Thus, as a cut-off value for AA, we used a ΔAge of ≥ 5 years, indicative of on average an overestimation by 5 years across all models. Similarly, for DA we used a ΔAge of ≤ −5 years, indicative of on average an underestimation of the chronological age by 5 years across all models. For CA, we used a cutoff of |ΔAge| < 2 years.

To identify which APs were significantly associated to ΔAge, we fitted linear models for each AP individually. Across both datasets, we used the following models:

*Arthur et al., 2021*— Protein expression ∼ Age+Gender+BMI+ ΔAge

*Robbins et al., 2021*— Protein expression ∼ Age+Gender+ BMI+Ethnicity+ΔAge

We extracted the *p*-values and estimates of our variable *ΔAge* to obtain the estimated association with the protein level, while statistically correcting for the other demographic variables. Nominal *p*-values were adjusted using an FDR correction (Benjamini-Hochberg) and proteins were considered differentially expressed at an adjusted *p*-value < .05.

To identify which APs show significant differences between our identified groups in terms of relative expression, we fitted linear models for each protein individually while including our ‘Biological Age Group’ (AA, DA or CA) as categorical variable. Across both datasets, we used the following models:

*Arthur et al., 2021*— Protein expression ∼ Age+Gender+ BMI+Biologial Age Group

*Robbins et al., 2021*— Protein expression ∼ Age+Gender+ BMI+Ethnicity+Biologial Age Group

We extracted the *p*-values and estimates of our categorical variable *Biological Age Group* to obtain the differences in relative expression levels and significance levels across all proteins, while statistically correcting for the other demographic variables. Nominal *p*-values were adjusted using an FDR correction (Benjamini-Hochberg) and proteins were considered differentially expressed at an adjusted *p*-value < .1.

### 2.11 Partial correlations for clinical blood values

To assess if ΔAge estimates may have clinical relevance, we obtained clinical blood values from Arthur et al. (2021) (Arthur et al., 2021). Prior to analyses, outliers in clinical blood variables, i.e., values 1.5 times the interquartile range (IQR) below the first quartile, or 1.5 times the IQR above the third quartile, were removed. We only excluded those scores within an individual that were considered outlier scores within a variable and included the remaining clinical blood values for these individuals. Partial correlations between ΔAge and clinical blood values were calculated while statistically correcting for age and BMI effects using the *pcor.test* function from the *ppcor* package (v1.1) in R.

## 3 Results

### 3.1 Identification of plasma proteins associated with aging

To come to a preserved human aging proteome, we tested which plasma protein expression levels were associated with age. To identify these proteins, we integrated the results of four independent, large-scaled studies on different cohorts using plasma proteome data of ∼5,000 proteins generated with the SOMAscan platform (Sathyan et al., 2020b; Arthur et al., 2021; Ferkingstad et al., 2021; Robbins et al., 2021). Despite clear differences between study cohorts, we obtained plasma proteomic information on approximately 5,000 proteins in individuals of the ages 16–95 years old across cohorts (Table 1).

**FIGURE 1:**
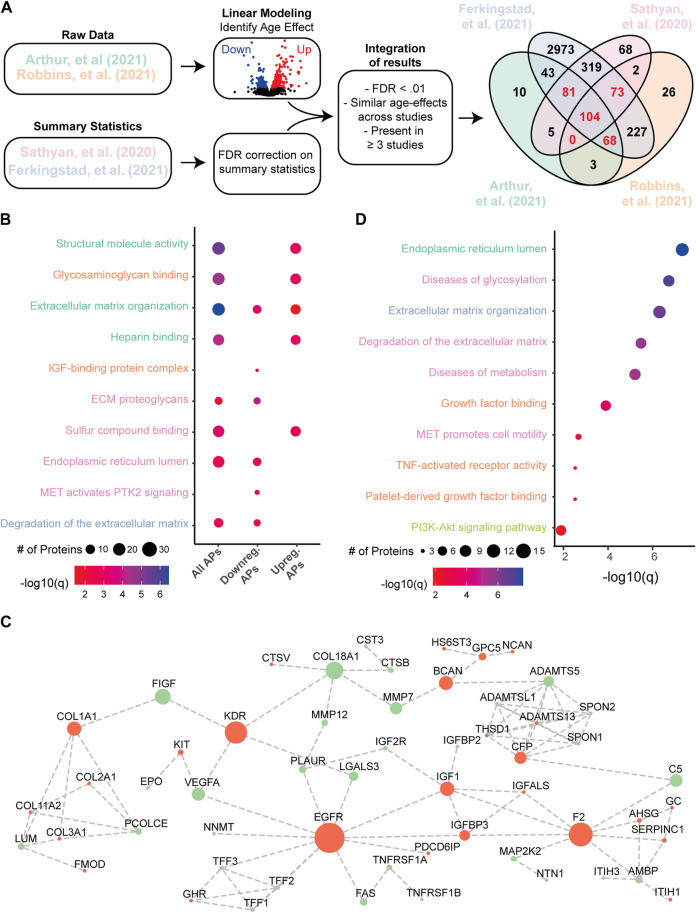
Identification of plasma proteins associated with aging. **(A)** Workflow and selection criteria used for the identification of APs. The Venn diagram depicts the results of the integration across datasets. Highlighted in red are 326 proteins with significant differences (FDR <.01) in ≥3 different studies, of which 273 APs showed similar age-associated effects across all studies. **(B)** Overview displaying a wide variety of significant functional enrichments of all APs, and the subsets of all downregulated APs and all upregulated APs. Dot size represents the number of proteins involved, and dot color represents significance levels as -log10(FDR). Terms are color coded based on database: Green = Gene Ontology (GO)-Cellular Component, Orange = GO-Molecular Function, Blue = GO-Biological Process, Pink = REACTOME database. A full overview is presented in Supplementary Table S4. **(C)** Visual representation of the protein-protein interaction (PPI) network of a highly interconnected subnetwork within all APs, consisting of 56 APs. Red nodes indicate decreased expression levels in the plasma during aging and green nodes indicate increased plasma expression levels during aging. Sizes of nodes reflect the number of connections to other proteins. **(D)** Overview displaying the various enriched terms of the PPI-subnetwork. Dot sizes and colors represent similar properties as described in panel **(B)**. Terms are color coded based on database as described in panel **(B)**, with the addition of light green for the KEGG database.

First, we identified within each cohort which protein expression levels were associated with aging across the individual studies (Figure 1A). Using linear modeling we identified 314 unique proteins to be significantly associated with age (q < .01) in the dataset of Arthur et al. (2021) (Arthur et al., 2021), and 503 unique proteins in the dataset of Robbins et al. (2021) (Robbins et al., 2021) (q < .01, Figure 1A). For the two studies who provided detailed summary statistics, we performed an FDR correction across all nominal *p*-values obtained per protein. In Sathyan et al. (2020), who used a comparable linear model approach, we identified 652 unique proteins to be significantly associated with age (q < .01). In Ferkingstad et al. (2021), who used a more complex random effects model to estimate age effects, we identified 3,888 unique proteins significantly associated with age (q < .01). Using this approach, in total 4,002 unique proteins - reflecting almost 80% of all measured proteins - were found to be significantly associated with age in at least one study. Across all four studies, 104 proteins (∼2%) were significantly associated with age (q < .01, Figure 1A). We also identified similar estimated age-associated effects across cohorts for proteins that showed significant differences in two studies (Supplementary Figure S1). The high number of unique proteins associated with aging across studies may be explained due to the variability between cohorts and driven by the high statistical power of Ferkingstad et al. (2021).

Next, we focused on a preserved human plasma aging proteome. We selected proteins significantly associated with age after correcting for multiple comparisons (q < .01) in at least three studies to allow for minor cohort differences, with similar effect directions (up- or downregulated with age) across all studies (Figure 1A). A total of 273 plasma proteins were found to meet these criteria, from now on referred to as “Aging Proteins” (APs; Figure 1A, see Supplementary Table S1 for a complete overview). Among all APs, 196 showed increasing levels during aging and 77 proteins showed decreasing levels. A comparison of these results across literature focusing on age-associated changes in the blood (serum or plasma) using other methods than the SOMAscan platform provided additional evidence for age-associated changes in 139 of these proteins, of which 132 proteins show similar age-associated effects in at least one other study (Supplementary Table S2).

We further tested if our APs were also associated with exceptional forms of aging. For this, we used the results from the independent study of Sebastiani et al. (2021) (Sebastiani et al., 2021), who used the SOMAlogic platform to measure the levels of serum proteins of centenarians (mean age 105 years) compared to healthy controls (mean age 79 years; Table 1). We identified 214 of all 273 APs in these results based on SomaID. Despite differences in the used biomaterial (plasma versus serum) and a unique population of extraordinary ages, 166 APs (78% of identified APs) showed significant changes in the group comparison of centenarians versus controls (FDR < .05). These results were in high concordance with our aging effects (e.g., increased in centenarians compared to controls, and increased plasma expression with aging) as only few APs showed opposite effect directions (Supplementary Figure S2; Supplementary Table S3). Similar results were found in the same study using a Mass Spectrometry approach (Sebastiani et al., 2021), although less APs were identified (Supplementary Table S3). These results indicate that our APs further change their expression levels at extreme ages in a similar direction as we described before and again underline the important role of APs in aging and their preservation across cohorts.

Functional enrichment analyses of all APs highlight various processes, such as those related to structural molecule activity, glycosaminoglycan binding and extracellular matrix organization (all FDR < .05; Figure 1B, see Supplementary Table S4 for a complete overview). Some differences in enriched terms were found between APs that positively or negatively associated with aging. For example, the terms ‘MET activates PTK2 signaling’ and ‘IGF binding protein complex’ are only enriched in APs downregulated with age (Figure 1B). These results suggest that multiple processes are affected in the aging process, leaving a robust signature in the human plasma proteome as identified across studies and methods.

To further test if our APs were biologically connected to each other we used the String Database and identified a significant enrichment of protein-protein interactions (PPI) among all our APs (*p* = 1.0e^−16^). Interestingly, 56 (20.5%) of our 273 APs were found to form a highly interconnected PPI subnetwork (Figure 1C). This subnetwork is enriched for a diversity of pathways, such as Growth factor binding, diseases of metabolism and TNF-activated receptor activity (all FDR < .05, Figure 1D). Altogether, these results highlight a preserved human aging proteomic signature in the plasma across independent studies and methods and suggest that a variety of biological processes are contributing to or are affected during aging.

### 3.2 Aging proteins follow multiple trajectories and are linked to a variety of biological processes

**FIGURE 2:**
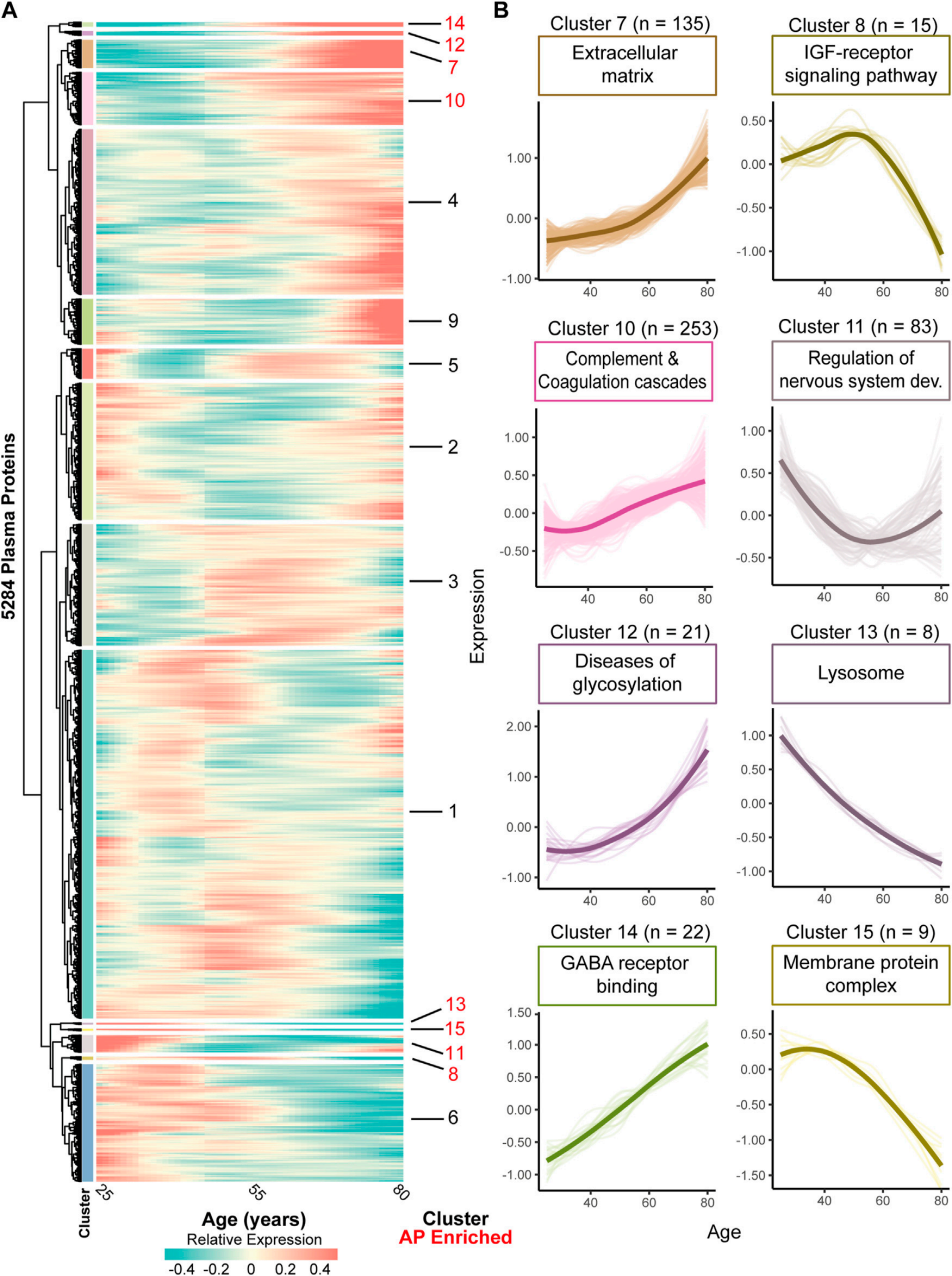
Clustering the complete plasma proteome on expression trajectories reveals AP enriched clusters involved in various functions. **(A)** Heatmap of identified clusters following unsupervised analyses of all plasma proteins based on expression trajectories across age using proteomic data of Arthur, et al. (Arthur et al., 2021) Color bars next to the dendrogram denote the individual clusters. Cluster numbers are marker red when enriched for APs using a hypergeometric test (q < .05). In the expression trajectory, blue colors indicate a relative decrease whereas red colors indicate a relative increase compared to the mean expression over time. **(B)** Zoom in of AP enriched clusters and the number of included proteins within each cluster as indicated by ‘n’. Thick lines represent the average expression trajectory of the cluster, and thin lines denote the trajectory of individual proteins. Above each cluster trajectory an example term of enriched function, molecular component, or pathway was included (all FDR <.05). For a complete overview of enriched GO-, KEGG- and Reactome terms within each cluster, see Supplementary Table S5.

Similar changes in expression levels over time across proteins may indicate their involvement in similar biological processes. Therefore, we clustered the complete plasma proteome on similar expression trajectories across the aging process. Using unsupervised hierarchical clustering on smoothened data (LOESS regression, see methods) of all 5,284 SOMAmers measured in Arthur et al. (2021) (Arthur et al., 2021) we identified 15 clusters with similar expression trajectories containing 8 (cluster 13) to 1772 (cluster 1) proteins (Figure 2A). Functional enrichment analyses of these clusters across the GO-, KEGG- and Reactome databases showed enrichments among a wide diversity of functions, cellular components, and pathways for these clusters, such as processes related to the extracellular matrix, regulation of neurogenesis, and complement and coagulation cascades (Supplementary Table S5). These results indicate that this approach may identify biologically connected networks across aging trajectories based on similar expression trajectories of all plasma proteins.

To test for processes robustly affected by aging we tested which clusters showed an overrepresentation of APs in the dataset of Arthur (Arthur et al., 2021). We found 8 clusters containing 8 to 253 proteins significantly enriched with APs (FDR < .05; Figure 2B, see Supplementary Table S6 with statistics and number of APs in each cluster). These clusters show a variety of expression trajectories during aging, such as linear and non-linear up- or downregulation, suggestive of respectively more or less involvement of these processes across the aging trajectory (Figure 2B). These clusters are enriched for several biological processes, including complement and coagulation cascades (cluster 10), the IGF-receptor signaling pathway (cluster 8) and processes related to the central nervous system development (cluster 11; Figure 2B, Supplementary Table S5). Largely we identified similar enriched processes among our clusters as in our initial analysis using only APs, but we also identified novel processes such as those related to the central nervous system, e.g., ‘regulation of nervous system development’ (cluster 11) and ‘GABA receptor binding’ (cluster 14), highlighting the added value of this network approach. Altogether, these results give more insight into age-associated processes and may provide a temporal insight across aging when these processes are affected or involved.

### 3.3 Aging proteins are strongly linked to age-associated diseases

As aging is a main risk factor in a multitude of diseases, we examined if our preserved APs are associated with phenotypes linked to diseases. For this we used summary statistics of the plasma protein associated phenotypes by Ferkingstad et al. (2021) in which they identified sets of plasma proteins associated with a variety of phenotypes after adjustment for sex and age effects. Of these phenotypes, 208 were established using a Case-Control approach, which we call disease-associated phenotypes, and 109 were measured quantitative traits (QTs).

**FIGURE 3:**
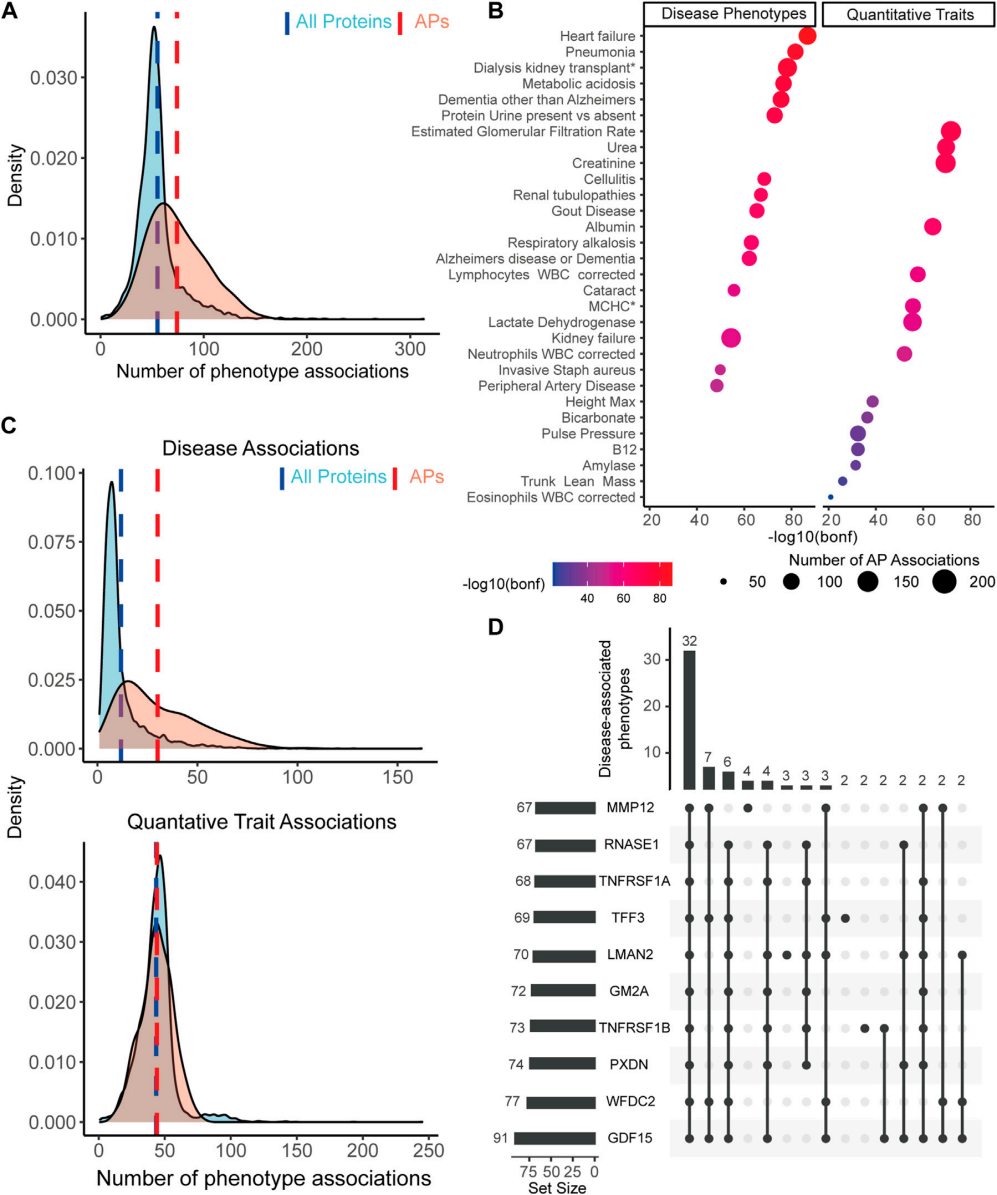
APs are more associated to disease-associated phenotypes than the rest of the plasma proteome. **(A)** APs (red distribution) show on average (red dashed line) significantly more associations across all included phenotypes than all other measured plasma proteins (blue distribution and dashed line). **(B)** Overview of the top 15 most AP enriched disease phenotypes and quantitative traits across 179 enriched phenotypes, ranked by Bonferroni corrected significance levels. Dot color represents the significance level on a log10(Bonferroni-adjusted *p*-value) scale. Dot size represents the number of APs associated to the corresponding phenotype. **(C)** When dividing all phenotypes based on disease phenotypes or QTs, we observe on average a significantly higher number of associations between APs and diseases compared to the rest of the plasma proteome (top distributions), but not between APs and QTs (bottom distributions). **(D)** Upset plot with an overview of the top 10 proteins associated with the most diseases, as reflected by the set size in the bar graph on the left side. Bar graph on top reflects the number of shared diseases across a subset of proteins, as illustrated by the dark dots represented below each graph. Numbers on top represent the number of shared phenotypes for the given subset of proteins.

We found that individual APs were associated to more phenotypes (on average 74 associations) compared to other plasma proteins (on average 55 associations, *p* < 2.2e-16, Figure 3A). Our top 20 APs with the most phenotype associations showed a high overlap with previous studies which used proteomic clocks to predict the phenotypes frailty, multimorbidity or mortality (Sathyan et al., 2020b; Tanaka et al., 2020) (Supplementary Table S7). We also detected several highly phenotype-associated novel proteins, namely, *WAP four-disulfide core domain protein 2* (WFDC2), *Trafficking protein particle complex subunit 3* (TRAPPC3), *Macrophage scavenger receptor types I and II* (MSR1) and *CUB domain-containing protein 1* (CDCP1). These results suggest that our APs may be more pleiotropic than other proteins, as reflected by their higher involvement in phenotypes.

To test which phenotypes were enriched with AP associations, we first determined the proportion of APs among all plasma proteins associated with each phenotype after adjustment for sex and age effects. Across all phenotypes, 179 were significantly enriched with APs (56%, Bonferroni corrected *p* < .01; see Supplementary Table S8 for a full overview), of which most were disease-associated (128 phenotypes). The majority of these still comprises diseases in which age is a known important risk factor, such as Heart Failure, Dementia and Renal Tubulopathies (Figure 3B). The most significant AP enriched QTs were *estimated glomerular filtration rate*, *urea* and *creatinine*, which may point to kidney functioning and perhaps liver functioning - two organs known to be affected during aging and important for maintaining homeostasis in the blood. Furthermore, several immune associated traits were found to be highly associated with APs, such as number of Lymphocytes, Neutrophils and Eosinophils. Together, these results point to a link between APs and age-associated diseases and may suggest impaired organ function and immune changes reflected in the blood.

To further establish the link between APs and diseases, we tested if APs are more specifically associated with disease-associated phenotypes and not QTs. To test this, we separated the phenotypes in their originally provided diseases or QTs category. In disease-associated phenotypes, we found on average more associations with APs (30 associations) than across all plasma proteins (12 associations; Welch’s *t*-test, *p* < 2.2e-16, Figure 3C). In QTs we identified no significant difference in the average number of associations across these subsets of proteins (both 44 associations; Welch’s *t*-test, *p* = .51, Figure 3C). These results underline an intriguing association between diseases and APs and suggest that APs are more affected by or involved in diseases.

To explore which APs are mostly associated with disease-associated phenotypes, we linked each individual AP to AP enriched disease-associated phenotypes. Leading our top 10 proteins we found *Growth/differentiation factor 15* (GDF15), a well described aging protein, and *Tumor necrosis factor receptor superfamily member 1A and 1B* (TNFRSF1A, TNFRSF1B), two receptors from the TNF-superfamily which are predominantly expressed by immune cells. GDF15 was associated with 91 phenotypes, which shared most phenotypes with other proteins from the top 10 (Figure 3D). Together, the top 10 shared a link to 32 phenotypes (Figure 3D), and the top 20 proteins still shared 21 phenotypes (Supplementary Figure S3). This suggests that a large group of APs plays a role across multiple shared diseases. Altogether, these results underline the link between age-associated plasma proteins and age-associated diseases and suggest the probable potential of these proteins in promoting the healthspan and reflecting health status due to their pleiotropic functioning and association to diseases.

### 3.4 A selection of aging proteins is a good predictor of chronological age

**FIGURE 4:**
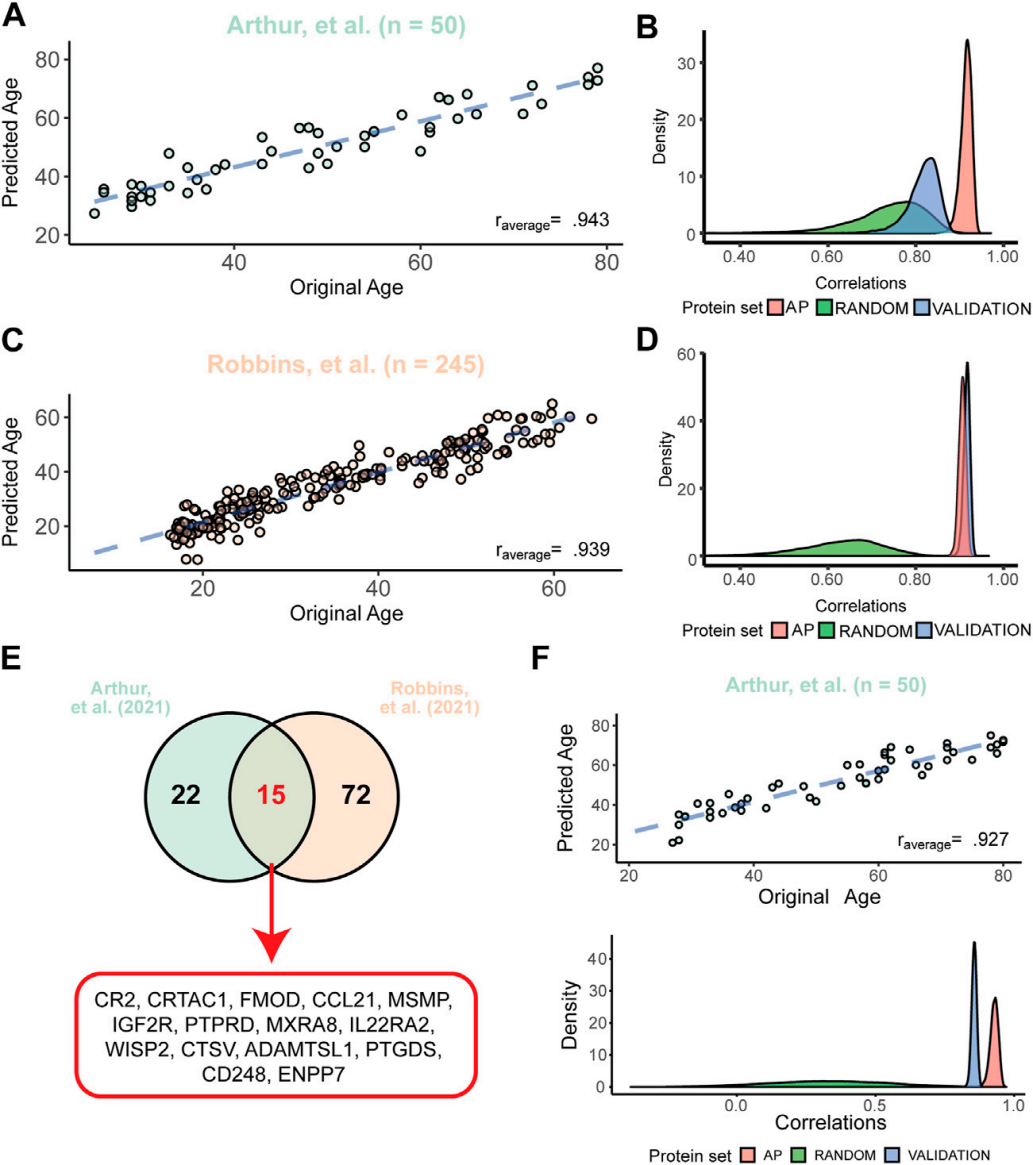
APs are better predictors of chronological age than other plasma proteins. **(A)** Example of the predicted versus the original age in the dataset of Arthur (Arthur et al., 2021) with the average correlation across 10,000 models. The blue dashed line depicts the fit after correction for the model bias. **(B)** Distributions of the 10,000 correlations between the original and the predicted ages obtained from models in Arthur (Arthur et al., 2021) using all 273 APs (“AP”), 273 random plasma proteins (“Random”) and the correlation of a model from one dataset tested in Robbins (Robbins et al., 2021) (“Validation”). **(C)** Example of the predicted versus the original age in the dataset of Robbins (Robbins et al., 2021) with the average correlation across 10,000 models. Dashed line is similar as described in panel **(A, D)** Similar distributions obtained from models created in the Robbins dataset (Robbins et al., 2021) as described in panel **(B)**. Here, the validation was the correlation of models created in Robbins (Robbins et al., 2021) and applied to Arthur (Arthur et al., 2021). **(E)** Overlap between the most used proteins across all 10,000 models for both datasets (used in >5,000 models), highlighting a signature of 15 APs used in the reduced models. **(F)** Example of the stable proteomic clock performance in the dataset of Arthur (Arthur et al., 2021) consisting of the 15 most used proteins as illustrated in panel **(E)**. Distributions below the correlation plot below illustrate the similar correlations obtained across all models as described in Panel **(B)** using the constant proteomic clock across all 10,000 models.

To test the predictive validity of our APs in predicting chronological age, we created multiple proteomic clocks based on our APs. First, we performed a LASSO regression analysis which allows for variable selection to predict ages using all 273 APs and sex as input variables. In short, for both datasets of Arthur (Arthur et al., 2021) and Robbins (Robbins et al., 2021), we repeated the LASSO regression analysis 10,000 times on ∼2/3rd of a randomly permuted sample to select the most important variables. Using these variables, we created predictive models and tested these models on the remaining sample by predicting their ages and then correlated the predicted ages to the original ages. On average across all 10,000 models, we obtained strong correlations between the original and predicted age in the datasets of Arthur (Arthur et al., 2021) (r_APavg_ = .943) (Figure 4A) and Robbins (Robbins et al., 2021) (r_APavg_ = .939, Figure 4C). Predictive models for Arthur (Arthur et al., 2021) consisted on average of 61 APs and 37 APs were used in the majority of the models (selected in >5000 LASSO regression analyses). For Robbins (Robbins et al., 2021) models consisted on average of 110 APs and 88 APs were used in the majority of models. These results show that AP expression levels in the plasma are good predictors of age.

To test if APs are better predictors of age than other plasma proteins, we questioned if models with similar sized sets of random proteins would perform equally well in the datasets as the models consisting only of APs. On average, our models using only APs highly outperformed models using sets of 273 random proteins before variable selection, and this effect was stronger in the dataset of Robbins (Robbins et al., 2021) (r_random_ = .662) than Arthur (Arthur et al., 2021) (r_random_ = .767) (Figure 4B; Figure 4D). Next, we tested the predictive validity of these models across datasets. To do so, we aimed to validate these models between datasets by applying the models created in the Arthur dataset (Arthur et al., 2021) to the Robbins dataset (Robbins et al., 2021) and *vice versa*, without recalibrating the models between datasets. When applying the models generated in the Arthur dataset (Arthur et al., 2021) to the Robbins dataset (Robbins et al., 2021), we observed a lower, but still strong correlation between predicted and chronological age (r_validation_ = .846; Figure 4B). The other way around, when applying the created models using the Robbins dataset (Robbins et al., 2021) to the Arthur dataset (Arthur et al., 2021), we obtained an even stronger predictive validity than in the original dataset (r_validation_ = .950; Figure 4D). These results highlight that our preserved AP plasma signature is more strongly associated with aging compared to other proteins.

With the aim to select APs with the most age predicting values, we overlapped the proteins used in the majority of the models for age prediction in Arthur (Arthur et al., 2021) (37 APs) or Robbins (Robbins et al., 2021) (87 APs). This resulted in 15 APs most used across both studies (Figure 4E). Using only these 15 APs, we repeated our age prediction analyses as described above using a Ridge regression to include all APs, thereby creating a single, constant proteomic clock. This reduced subset of APs still had a strong predictive validity across 10,000 models in the dataset of Arthur (Arthur et al., 2021) (r_APavg_ = .927, Figure 4F) and Robbins (Robbins et al., 2021) (r_APavg_ = .888). For both datasets similar sized sets of random proteins were on average poor predictors of chronological age (r_randomArthur_ = .324, r_randomRobbins_ = .323). Models built in the dataset of Arthur (Arthur et al., 2021) were on average still good in predicting age in the dataset of Robbins (Robbins et al., 2021) (r_validation_ = .855), and models build in the dataset of Robbins (Robbins et al., 2021) outperformed themselves in the dataset of Arthur (Arthur et al., 2021) (r_validation_ = .923). Together, these results show that our proteomic clock consisting of 15 APs is a very good predictor of chronological age, even across independent datasets.

To further corroborate the predictive validity of these proteomic clocks we aimed to test them in an independent cohort, i.e., a cohort not used for the selection of our APs. Since studies using similar proteomic methods, experimental designs and sharing of data are very limited, we decided to test our models on the small dataset provided by the COVIDome study (Sullivan et al., 2021) (Table 1). Although this dataset may not be a perfect control as it contained hospitalized patients, we focused on the 29 COVID-negative patients of which proteomic data was available. These patients ranged from 22 to 80 years and 268 of our APs were measured. After applying the models containing all APs on this dataset, we obtained on average a strong correlation for the models created in Robbins, et al. (Robbins et al., 2021) (r = .751) and moderate correlation in Arthur, et al. (Arthur et al., 2021) (r = .560) (Supplementary Figure S4). Applying the reduced models using the previously selected 15 APs showed a lower, but moderate correlation using the models of Robbins, et al. (Robbins et al., 2021) (r = .641), and similar results using models of Arthur, et al. (Arthur et al., 2021) (r = .523). Across all four sets of models we obtained on average no correlation (all average r ≈ 0) after permuting the model weights across APs, suggesting that our trained models still contains age predictive information in this independent cohort. Altogether, these results underline that our preserved AP signature has predictive information on aging across cohorts.

### 3.5 Assessment of altered blood signatures across aging trajectories

**FIGURE 5:**
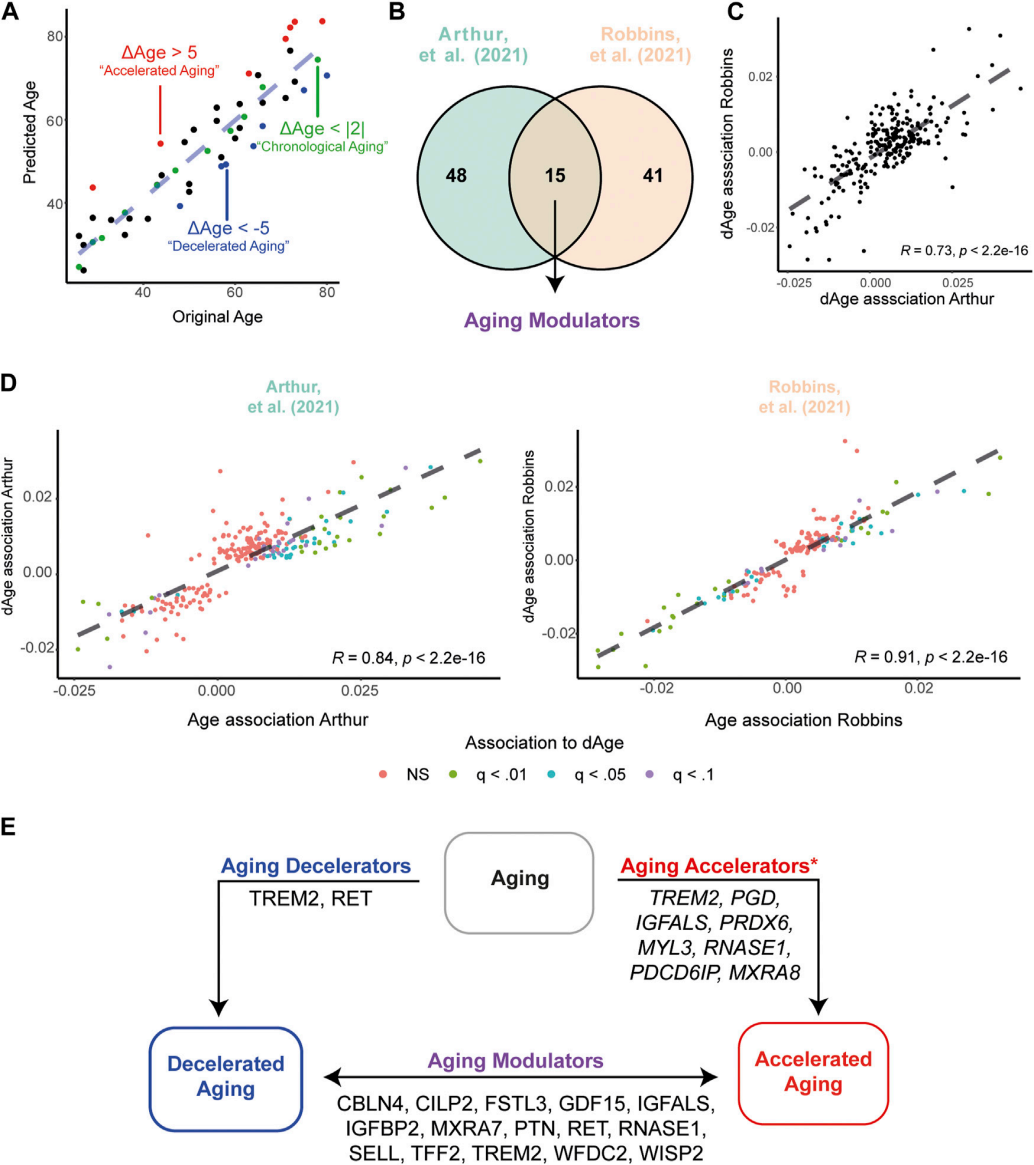
Accelerated and Decelerated agers show different expression levels of APs. **(A)** Example illustrating the identification of Chronological Agers (CA, green), Decelerated Agers (DA, blue) and Accelerated Agers (AA, red) based on the average ΔAge estimates using 273 APs. **(B)** Overview of number of significant associations between ΔAge and AP expression levels in the Arthur (Arthur et al., 2021) and Robbins (Robbins et al., 2021) dataset. **(C)** Associations between ΔAge and AP expression are highly similar between datasets. **(D)** Associations between ΔAge and AP expression are highly similar compared to the previously identified age-associations within the datasets. **(E)** Graphical summary of the integration of all results across datasets, pointing to proteins which may have important influences on the aging process. Proteins depicted in Aging Decelerators and Aging Modulators are the overlap of differentially expressed proteins between datasets. *For Aging Accelerators, all proteins are found to be significant (q < .01) in the CA versus AA comparison in the dataset of Robbins (Robbins et al., 2021) only. For a full overview of differentially expressed APs between biological age groups, see Supplementary Table S10,11.

When comparing the average predicted age of individuals based on the 273 APs with their chronological age, some individuals are either predicted older or younger based on their plasma profile (ΔAge, Figure 5A). Across the datasets of Arthur (Arthur et al., 2021) and Robbins (Robbins et al., 2021), we observed approximately normally distributed ranges of ΔAge values among both datasets (Shapiro-Wilk test; p_Arthur_ = .401, p_Robbins_ = .308) and no associations between chronological age and ΔAge was observed across both datasets (Supplementary Figure S5). The mean ΔAge in the dataset of Arthur (Arthur et al., 2021) was −0.01, with a mean average error of 3.955 years. In the Robbins dataset (Robbins et al., 2021), the mean ΔAge was 0.046, with a mean average error of 3.391 years. Altogether, these model statistics suggest an unbiased and reliable estimate of ΔAge across both datasets.

In our previous analyses, we highlighted a link between APs and phenotypes related to kidney and liver functioning (Figure 3B). To further explore if ΔAge may be informative for health status, we explored if our ΔAge estimates associate with clinical blood markers (CBMs). CBMs, such as levels of cholesterol and CO_2_, are used to assess a general state of health or how well certain organs are working. Arthur et al. (2021) (Arthur et al., 2021) provides extensive blood phenotyping including a clinical and immunological panel of 116 male individuals of which they also obtained plasma proteomic data. We obtained several nominal significant partial correlations between ΔAge and CBMs after correcting for chronological age and BMI. For example, higher levels of uric acid were associated with a higher (biologically older) ΔAge (*Partial r* = .267, *p* = .005), whereas higher levels of magnesium were associated with a lower (biologically younger) ΔAge (*Partial r* = −.197, *p* = .036). Near significant associations were found for levels of aspartate aminotransferase, a marker of liver functioning, (*Partial r* = −.189, *p* = .051) and HDL cholesterol, sometimes referred to as the good cholesterol (*Partial r* = −.171, *p* = .072). For a full overview, see Supplementary Table S9. These results suggest that ΔAge estimates may potentially have clinical relevance in assessing a general state of health.

Next, we questioned which APs contribute the most in observed deviations between chronological and biological ages. To do so, we applied two statistical modeling approaches. First, we associated AP expression levels to ΔAge estimates, while statistically correcting for chronological age, sex, BMI and ethnicity (latter variable in the dataset of Robbins (Robbins et al., 2021) only). These associations would identify potential ‘aging modulators’ across our cohorts. We identified 63 APs to be significantly associated with ΔAge in the Arthur dataset (Arthur et al., 2021) and 56 in the Robbins dataset (Robbins et al., 2021), of which 15 APs overlapped (all FDR < .05; Figure 5B). For example, we identified positive associations between GDF15 and ΔAge, suggesting that higher levels of this protein in the plasma may reflect to a higher biological age (‘accelerated’ aging, AA), whereas lower levels may reflect to a lower biological age (‘decelerated’ aging, DA). Associations between APs and ΔAge showed high concordance between datasets (r = .73, *p* < 2.2e-16; Figure 5C), suggesting similar contributions in modulating aging trajectories across cohorts. Moreover, the associations between APs and ΔAge showed similar effects compared to initially identified aging effects in the dataset of Arthur (Arthur et al., 2021) (r = .86, *p* < 2.2e-16) and Robbins (Robbins et al., 2021) (r = .91, *p* < 2.2e-16; Figure 5D). Together, this suggests that the AP signature in the plasma of decelerated agers reflects a more youthful systemic environment, whereas accelerated agers show an older systemic environment.

As processes and proteins contributing to AA and DA may differ, we aimed to define the proteins contributing to decelerated or accelerated aging. Therefore, as our second approach, we defined three biologically aging groups: 1) Chronological Agers (CA), with a maximum of 2 years difference between estimated age and chronological age (|ΔAge| < 2), 2) Decelerated Agers, with an underestimation in their age based on plasma proteomic profile (ΔAge < −5), and 3) Accelerated Agers, with an overestimation in their age based on proteomic profile (ΔAge >5); Figure 5A). In the Arthur dataset (Arthur et al., 2021) 46 individuals showed CA, 25 DA with a maximum underestimation of more than 11.5 years, and 28 AA with a maximum overestimate of almost 12 years. The dataset of Robbins et al. (Robbins et al., 2021) contained 255 individuals showing CA, 88 DA with a maximum underestimate of more than 16 years, and 92 AA with a maximum overestimate of almost 13 years. For complete demographics of these subsamples, see Table 2. No significant differences in demographics were found between groups, except for BMI in Robbins et al. (Robbins et al., 2021) in which the AA group had on average the highest BMI measures and the DA group the lowest. These results hint to an association between BMI and aging quality.

**TABLE 2:** Description of altered aging trajectory groups. Provided are the number of individuals per group, chronological age, the discrepancy of estimated proteomic and chronological age, and the demographics of the individuals. *P*-values indicate if there are differences for the given variables between the groups.

	Arthur et al., 2021				Robbins et al., 2021			
	Accelerated (n = 28)	Chronological (n = 46)	Decelerated (n = 25)	p	Accelerated (n = 92)	Chronological (n = 255)	Decelerated (n = 88)	p
Age (mean; sd [range])	50.29; 17.79 [26–79]	47.59;16.87 [25–80]	53.04; 15.00 [26–77]	.416	35.73; 12.09 [16.7–59.80]	33.64;12.64 [16.7–65.2]	34.10; 16.81 [17.00–65.90]	.441
ΔAge (mean; sd [range])	6.98; 1.90 [5.03–11.93]	−0.28;1.06 [-1.99–1.76]	−6.97;1.76 [-11.66–-5.09]	<.001*	7.06; 1.76 [5.03–12.93]	0.11;1.11 (−1.99–1.99)	−7.27;2.29 [-16.37–-5.02]	<.001*
Gender (m/f)	23/5	36/10	23/2		43/49	117/138	39/49	
BMI (mean; sd [range])	25.57; 2.92 [17.8–29.5]	24.62;2.59 [18.5–29.3]	24.75; 3.13 [20.3–30.0]	.357	28.88; 6.62 [18.03–50.94]	26.39;5.27 [17.31–48.26]	24.65; 5.16 [17.30–39.29]	<.001*
Race (black/caucasian)	-	-	-		29/63	105/150	37/51	

Note: *p*-values calculated across groups using a one-way ANOVA.

Next, we compared our DA groups against our CA groups to identify a proteomic signature of decelerated aging, while statistically correcting for chronological age, sex, BMI and ethnicity (latter variable in the dataset of Robbins (Robbins et al., 2021) only). Between DA and CA nine proteins were differentially expressed (FDR < .1) in the dataset of Arthur (Arthur et al., 2021) (Supplementary Table S10), and 11 proteins in Robbins (Robbins et al., 2021) (Supplementary Table S11). Only two proteins overlapped between datasets, namely, *Proto-oncogene tyrosine-protein kinase receptor Ret* (RET) and *Triggering receptor expressed on myeloid cells 2* (TREM2), so called ‘aging decelerators’ (Figure 5E). When repeating this for AA compared to CA, in the dataset of Arthur (Arthur et al., 2021) no differentially expressed proteins were found (Supplementary Table S10). Conversely, in the Robbins dataset (Robbins et al., 2021), 46 proteins were differentially expressed between AA and CA (FDR <.1, Supplementary Table S11). Expression of the four proteins that overlap between DA versus CA and AA versus CA comparisons in this dataset – namely, *Chondroadherin* (CHAD), *Complement receptor type 2* (CR2), *Protein SET* (SET), and TREM2 - go in opposite directions in DA compared to AA. Altogether, these results highlight several proteins which may in multiple ways be associated with the aging process, either by accelerating or decelerating the process, or play a modulatory role in aging which may have divergent effects depending on the expression levels (Figure 5E).

## 4 Discussion

The aim of this study was to identify markers of aging and potential targets to improve the quality of aging. By integrating the results of four independent large-scaled human plasma proteome datasets, we identified a set of 273 preserved aging proteins (APs) with similar age-associated effects across studies, enriched for a variety of functional processes and highly associated with a multitude of age-associated phenotypes. Using only 15 of these proteins we were still able to estimate an individuals’ age with good accuracy, emphasizing their potential as biomarkers of aging. Moreover, we identified a subset of proteins differentially expressed in individuals with a plasma proteomic profile that diverged from their chronological age, which may be valuable targets to improve the quality of aging.

Across the four integrated studies, almost 80% of all measured plasma proteins (> 4,000) was associated with aging across studies, illustrating the complexity and variability of the aging process. This number exceeds a previous systematic review of age-associated proteins across tissues and cells (Johnson et al., 2020). This results of the higher throughput of our used datasets allowing a broader, more specific screening of the aging plasma proteome than before, but also because of the inclusion of bigger cohorts such as the deCODE cohort. Illustrative of this first benefit is highlighted in our phenotype associations, in which we identified several novel cross-disease associated proteins. Although we focus only on a small subset of this large group of APs (∼2%), we show in our models that these 273 APs are more associated to aging than other proteins in the plasma. Moreover, the age-predictive validity of our clocks is close to previously reported aging clocks using 491 age-associated proteins (Lehallier et al., 2020), even in our reduced models using 15 APs. Although these presented proteins may be different compared to other presented proteomic clocks (Tanaka et al., 2018; Lehallier et al., 2019; Sathyan et al., 2020b), this can be explained due to a variety of factors. Across studies there may be several technical factors, such as used anti-coagulants, and biological differences, such as different age ranges, ethnicity and corrections for BMI, which may influence the plasma proteome in the cohorts. To overcome these differences, we therefore focused on the overlap between the different studies as they also present several of these confounding factors.

A limitation of our selection strategy is a potential bias in selection due to the single platform used across studies. Nevertheless, direct comparisons of the SOMAscan and Olink platforms have shown mostly moderate to high correlations between measurements (Ferkingstad et al., 2021; Haslam et al., 2022; Katz et al., 2022). It is important to note that different normalization procedures, inter- and intraplate variability as observed in the SOMAscan assay may affect our selection (Candia et al., 2017), however we have tried to overcome this by finding the overlap across studies. Lastly, we presented additional evidence for 132 APs, which are preserved across different methods (Supplementary Table S2). Furthermore, it must be noted that this preserved signature includes information across individuals of different ethnicities, namely, white and black Americans, Jewish Ashkenazi and an Icelandic population. Importantly, our analysis across the dataset of Robbins et al. (Robbins et al., 2021) showed more than 1,000 significant differences in protein expression levels between ethnicities of which 138 APs (51% of all APs), suggestive of altered aging trajectories across diverse genetic backgrounds (Supplementary Figure S6). In line with this, we want to highlight the limitation of the cross-sectional nature of this data. Given the high variability in the plasma proteome and the strong link between APs and diseases, this might impact our identified aging proteome. Longitudinal measurements are essential to disentangle APs from other confounding factors such as age-associated diseases, and to identify protective and detrimental proteins across aging trajectories. This leaves a great opportunity for future research to better disentangle the human aging proteomic signature.

Among our APs we identified a subset of well-known candidates such as GDF15, a stress responsive cytokine resulting of mitochondrial dysfunction (Conte et al., 2022). GDF15 is described as a key player in human aging and predictor of mortality (Wiklund et al., 2010; Johnson et al., 2020; Conte et al., 2022). Using the Human Ageing Genomic Resources database (Tacutu et al., 2018), we verified that 41 APs had either genetic variants associated with longevity, were known to induce or inhibit cellular senescence or were altered in a dietary restriction intervention resulting in delayed age-related degeneration or lifespan expansion across different species (Tacutu et al., 2018). Moreover, several identified proteins, such as *Insulin-like growth factor-binding protein 2* (IGFBP2) and *Insulin-like growth factor-1* (IGF1), belonged to the IGF family and are part of a highly conserved pathway which has been shown to play a major role in previous aging and longevity studies across species (Barbieri et al., 2003; Rincon et al., 2005). Together, these findings complement to previous well described results of proteins and pathways related to aging and longevity and highlight the most potential therapeutic targets to improve the aging process.

Additionally, we show an association between APs and their involvement in disease. Even after initial correction for age effects in associations between plasma levels and phenotypes, our results indicate that our set of APs are more associated with phenotypes, specifically disease-associated, than the rest of the plasma proteome. While this should be interpreted carefully as no causal evidence is provided between plasma levels and phenotypes, we believe that the combination of our results suggests that targeting age-associated proteins in the plasma may be of value to combat age-associated diseases and thereby increases the healthspan. Likewise, it has been suggested that increasing the healthspan may be the most important treatment for age-associated diseases and several interventions with translational potential have been put forward to reach this goal (Kaeberlein et al., 2015). Importantly, targeting these proteins systemically will require more elaborate studies to avoid unnecessary side-effects or identify underlying mechanisms. For example, increasing the AP *Beta-2-microglublin* systemically has adverse effects on hippocampal-dependent cognitive functioning and neurogenesis (Smith et al., 2015). Conversely, a cleaved domain of the AP *Klotho,* KL1, was shown to be sufficient to enhance cognition and to mediate the effects of the complete protein (Gupta et al., 2022).

Previous research already indicated how a ‘younger’ systemic environment contributes to slowing the aging process or even to rejuvenation of tissues across the body (Villeda et al., 2011; Loffredo et al., 2013; Katsimpardi et al., 2014; Sinha et al., 2014; Villeda et al., 2014; Baht et al., 2015; Castellano et al., 2017; Huang et al., 2018; Palovics et al., 2022; Yankova et al., 2022). In line with these findings, our results show altered expression levels of APs between those who deviate from chronological aging and indicate a more aged systemic environment in those who show proteomic signs of accelerated aging. It is important to emphasize that although these results reflect altered aging signatures in the plasma proteome, these results are based on a relatively small number of proteins given the size of the full proteome. Other unmeasured proteins may play a confounding or mediating role in altering aging trajectories. Interestingly, we also identified that changes in several clinical health biomarkers associated with differences between proteomic and chronological age. We found an association with higher uric acid levels, which is associated with impaired kidney functioning (Bellomo et al., 2010), in addition to an enrichment of AP associations in known markers used to assess kidney functioning (estimated glomerular filtration rates, urea and creatinine). Furthermore, both uric acid and urea are mainly synthesized in the liver (Morris, 2002; El Ridi and Tallima, 2017), and uric acid has been hypothesized to contribute to the increased life span of humans compared to other mammals (Alvarez-Lario and Macarron-Vicente, 2010). Together, this may point to these organs being highly affected by or vulnerable to aging. As these two organs are involved in filtering the blood, it may be that due to the aging process these organs cannot properly function anymore, leading to a more toxic and less homeostatic environment in the blood over time. A recent study using umbilical cord plasma injections in human elderly showed beneficial effects on estimated ages using epigenetic profiles, but also improved biomarkers of kidney functioning such as creatinine levels and estimated glomerular filtration rates (Clement et al., 2022). Altogether, this points to an important role of these organs in aging and suggests impaired functioning of these organs over time. This may ultimately cause homeostatic disturbances in the blood, leading to detrimental biological events across the body.

Besides these two organs, we also want to highlight the presence of an age-associated plasma signature of brain aging. We found both dementia-related phenotypes and a cluster related to central nervous system development to be enriched for AP associations. Moreover, we identified an important role for TREM2, of which several genetic variants are a well-known risk factor for Alzheimer’s disease and other neurodegenerative diseases (Carmona et al., 2018), in individuals who deviate from their chronological age. Given the strong links between age-related cognitive decline, dementia and aging, these potential targets may provide valuable insights in brain aging. Targeting this may both reduce a burden on caretakers, and increase autonomy of individuals on later ages, ultimately leading to an increased healthspan.

In summary, we presented a preserved human proteomic signature of aging which appears to be linked to age-associated diseases. Using this preserved aging signature, we provide insights in some of the most important pathways affected during the aging process. Altogether, these results contribute to the understanding of aging and put several important proteins and mechanisms forward, which may be of use for further studies to experimentally disentangle the biological mechanisms of aging.

## Data Availability

The original contributions presented in the study are included in the article/Supplementary Material, further inquiries can be directed to the corresponding author.
