# Supplementary material for: Markers of aging: Unsupervised integrated analyses of the human plasma proteome
Source: Front Aging. 2023 Feb 22;4:1112109. doi: 10.3389/fragi.2023.1112109 (PMC9992741; doi:10.3389/fragi.2023.1112109)
Supplement: Supplementary file 1 [file Table2.DOCX]

Supplementary Material

Markers of Aging: Unsupervised Integrated Analyses of the Human Plasma Proteome

L. Coenen^1,2^, B. Lehallier^3^, H.E. de Vries^2^, J. Middeldorp^1*^

^1^Department of Neurobiology and Aging, Biomedical Primate Research Centre, Rijswijk, The Netherlands.

^2^Department of Molecular Cell Biology and Immunology, Amsterdam UMC location Vrije Universiteit Amsterdam, Amsterdam Neuroscience, Amsterdam, The Netherlands.

^3^Alkahest Inc, San Carlos, California, USA.

# Supplementary Figures and Tables

See attached excel sheet for supplemental tables.

## Supplementary Figures

**Supplemental Figure 1.** Estimated age-associated effects of APs are similar across independent datasets. Heatmap displaying estimated age effects of 925 unique proteins associated with age as identified in at two studies showing highly similar estimated effects across different cohorts. Proteins are sorted based on age effects as identified in Ferkingstad, et al. (2021) with the most positive associations, e.g. most increased expression during aging, in the top rows and most negative associations, e.g. strongest decreased expression during aging, on the bottom rows. Each row indicates a single protein. Red colors indicate positive association with age in linear models, blue colors indicate negative associations with age and grey color reflects the absence of an age-associated effect in the given cohort for the protein.


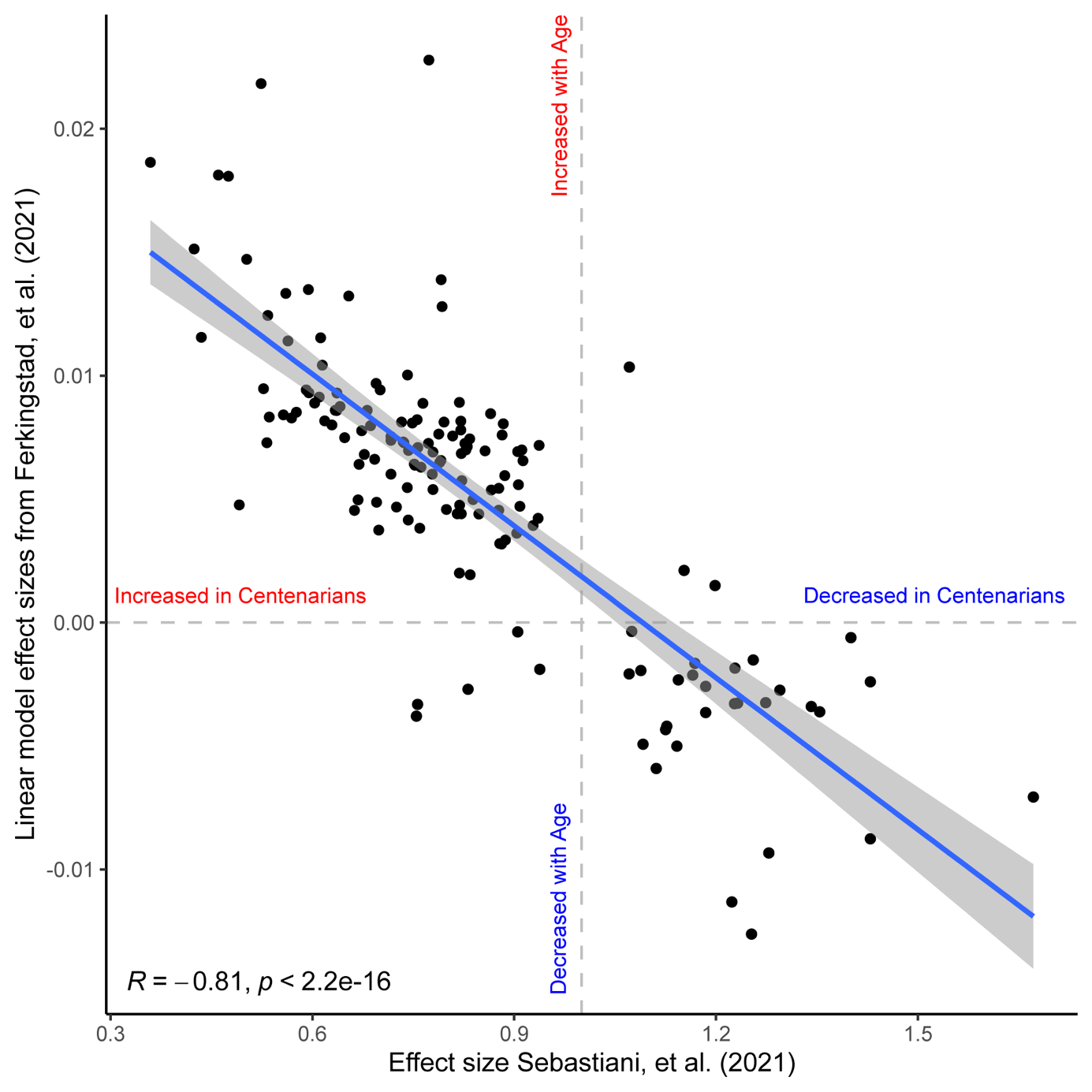


**Supplemental Figure 2.** APs differentially expressed between Centenarians and younger controls show concordant age-associated effects as identified in Ferkingstad, et al (2021). Comparison of AP age-associated effects in Ferkingstad, et al. (2021) and in Sebastiani, et al. (2021) who identified expression differences in controls (mean age 79 years) compared to centenarians (mean age 105 years), expressed as Fold Change (FC). FC values < 1 indicate an increase in centenarians and FC values > 1 indicate a decrease in centenarians. For the 166 APs with differential expression between Centenarians and controls, a highly significant association was found between estimated aging effect sizes.


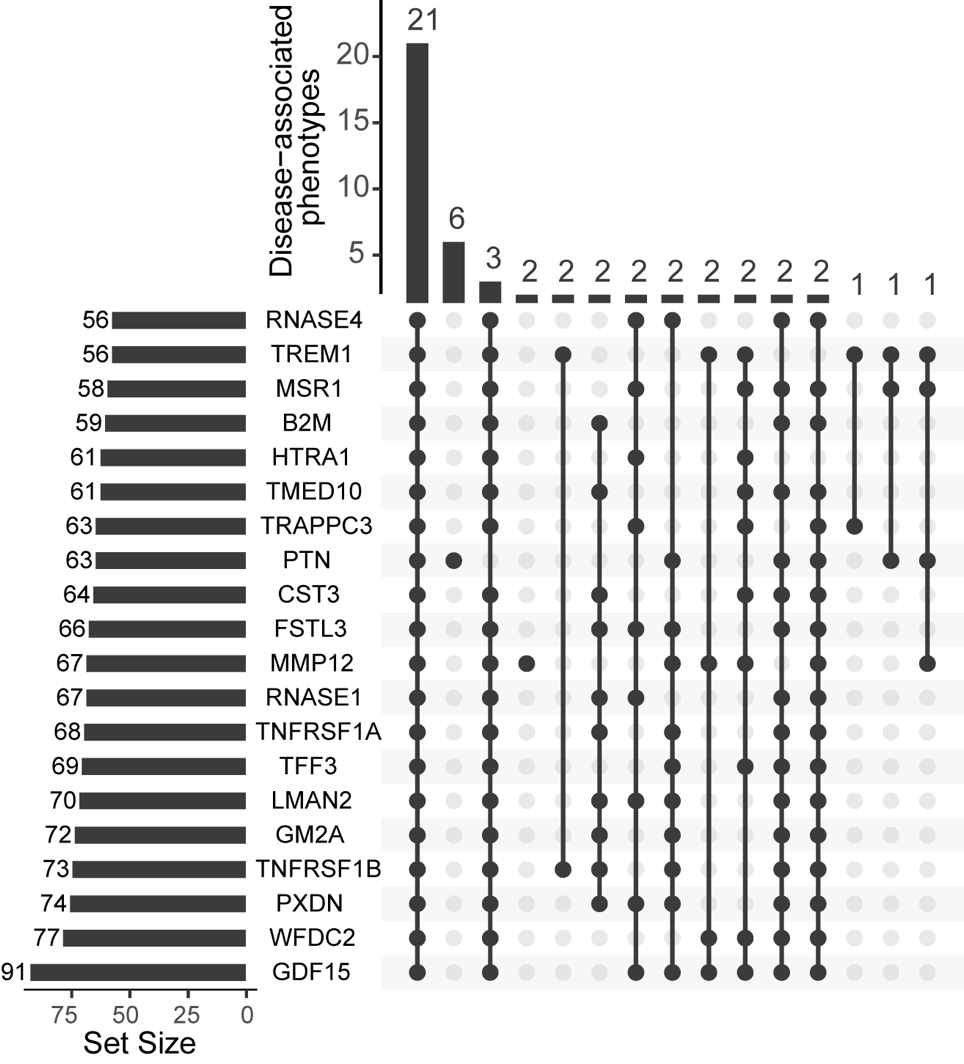


**Supplemental Figure 3.** Top 20 most disease-associated APs show similar involvement across disease-associated phenotypes. Bar graphs on the left side indicate the number of disease associations per AP. Bar graph on top reflects the number of shared diseases across a subset of proteins, as illustrated by the dark dots represented below the graph. Numbers on top represent the number of shared phenotypes for the given subset of proteins. This overview reveals a complex pattern of mostly shared APs associated with diseases.


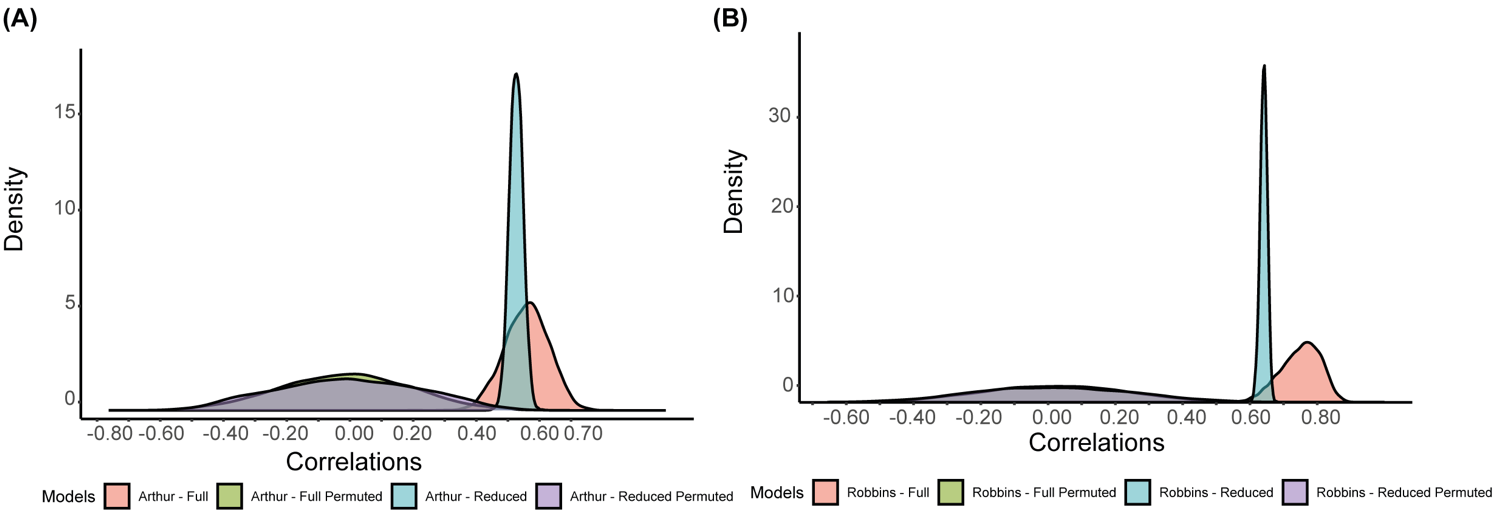


**Supplemental Figure 4.** Overview of the correlation distributions of the models using all APs (“Full”), the reduced models using the 15 most predictive APs (“Reduced”), and the same models with the weights of the models permuted (“Full Permuted” and “Reduced Permuted”) obtained in the datasets of **(A)** Arthur, et al. (2021) and **(B)** Robbins, et al. (2021) applied to the independent COVIDome dataset [42].

**
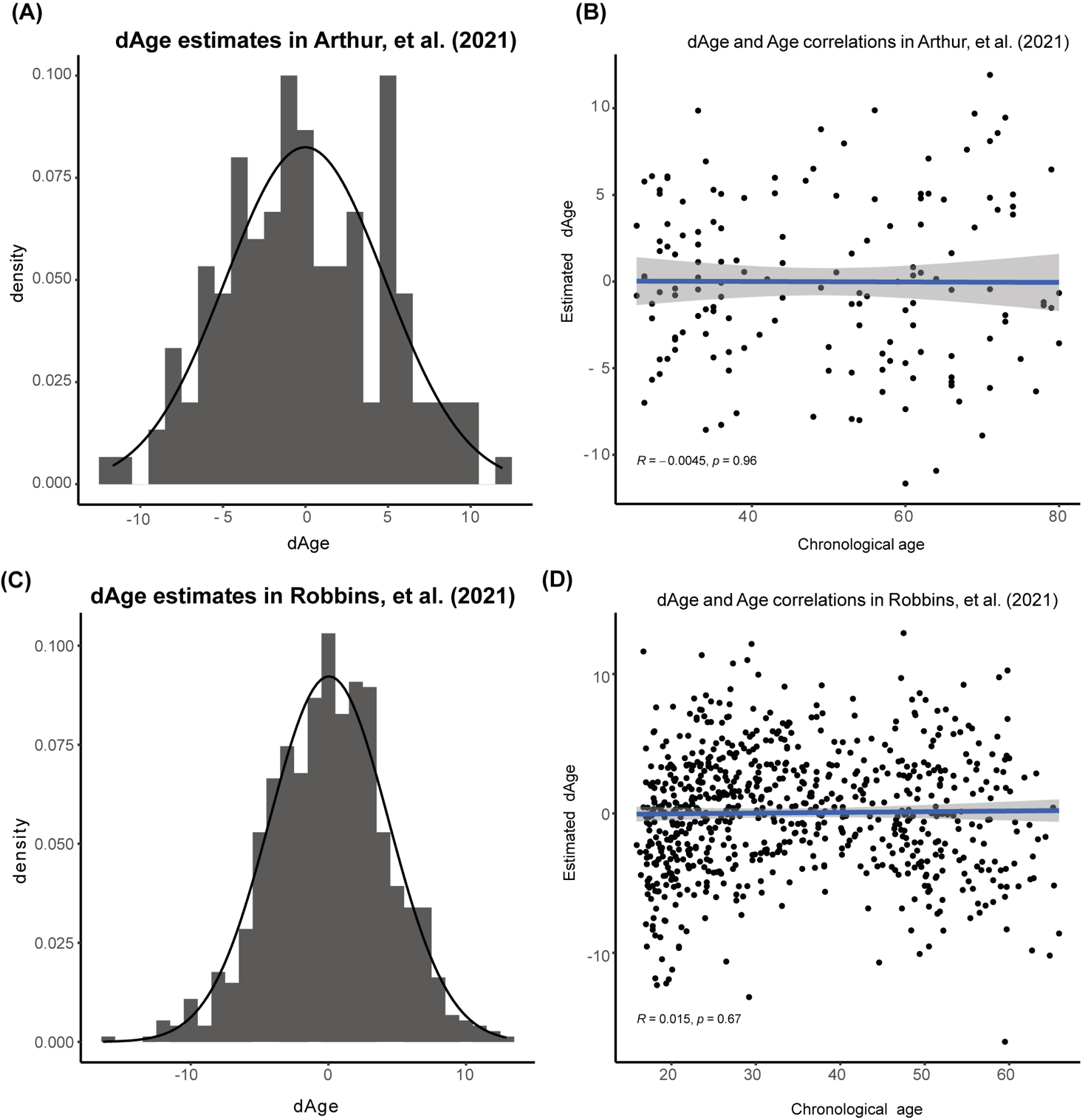
**

**Supplemental Figure 5.** The proteomic clocks consisting of APs are able to predict deviations between chronological and biological age unbiasedly. **(A)** Overview **of** ΔAge estimates in the dataset of Arthur, which is following a normal distribution. **(B)** Correlation plot showing that there is no association between chronological age and estimated ΔAge in the dataset of Arthur. **(C)** Overview of ΔAge estimates in the dataset of Robbins, showing a normal distribution. **(D)** No association was found between chronological ages and ΔAge estimates in the dataset of Robbins

**
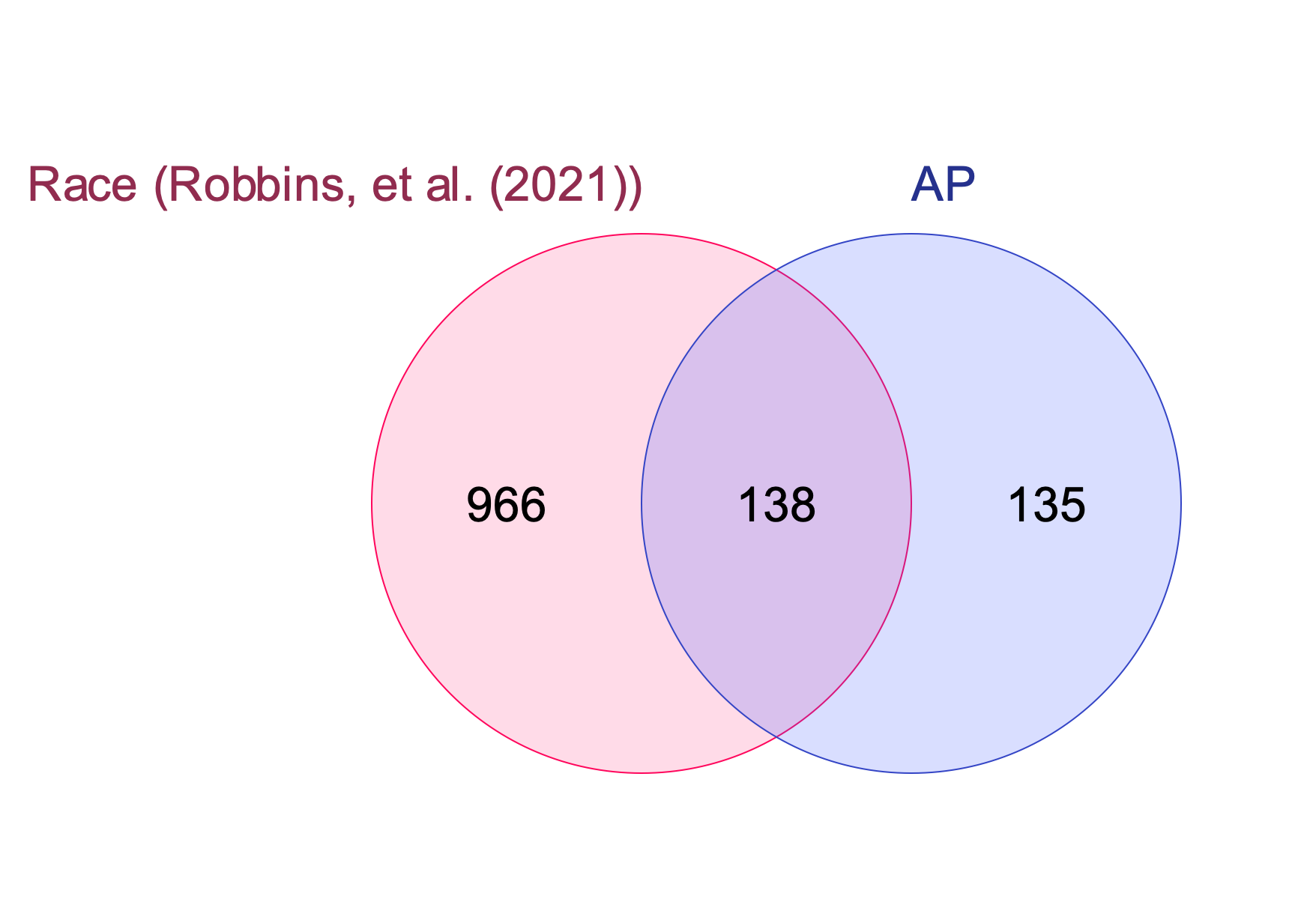
**

**Supplemental Figure 6.** Overview of the overlap differentially expressed plasma proteins between ethnicities (q < .01) in the linear models conducted on Robbins et al. (2021) and our defined APs. More than 50% of APs are differentially expressed in those with a non-Caucasian background.
